# Elevated Serum Uric Acid is Associated With Poor Survival in Advanced HCC Patients and Febuxostat Improves Prognosis in HCC Rats

**DOI:** 10.3389/fphar.2021.778890

**Published:** 2021-11-11

**Authors:** Le Wu, Wenlong Yang, Yu Zhang, Xiaoyue Du, Nan Jin, Wen Chen, Huangbao Li, Shouhua Zhang, Baogang Xie

**Affiliations:** ^1^ Department of Pharmaceutics, Medical College of Jiaxing University, Jiaxing, China; ^2^ School of Pharmaceutical Science, Nanchang University, Nanchang, China; ^3^ Department of Infectious Diseases, Second Affiliated Hospital of Nanchang University, Nanchang, China; ^4^ Department of Breast Surgery, Jiangxi Provincial Cancer Hospital, Nanchang, China; ^5^ Department of Hepatobiliary and Pancreatic Surgery, The Affiliated Hospital of Jiaxing University, Jiaxing, China; ^6^ Department of General Surgery, Jiangxi Provincial Children’s Hospital, Nanchang, China

**Keywords:** advanced HCC patients, serum uric acid, survival time, febuxostat, prognosis

## Abstract

**Objective:** Serum uric acid is associated with tumor progression and hepatocarcinogenesis. Here, we aimed to determine whether serum uric acid is related to the survival time of patients with hepatocellular carcinoma (HCC) and whether the inhibition of uric acid production affects the progression and survival of rats with HCC.

**Methods:** The follow-up data of 288 patients with advanced HCC were analyzed. Ten purine metabolites in serum and liver samples of diethylnitrosamine (DEN)-induced HCC rats were quantitatively determined by an established UPLC-MS/MS method. On this basis, febuxostat, a specific inhibitor of xanthine oxidase (XOD), was used to interfere with HCC rats.

**Results:** The serum uric acid level of HCC patients was significantly negatively correlated with survival days (*r* = -0.155). The median survival time was 133.5 days in the high uric acid group (>360 μmol/L, *n* = 80) and 176.0 days in the normal serum uric acid group (<360 μmol/L, *n* = 208, *p* = 0.0013). The levels of hypoxanthine, guanine, and uric acid; XOD activity; and xanthine dehydrogenase mRNA expression in the serum or liver samples of HCC rats were significantly upregulated compared with those in the control group. After febuxostat intervention in DEN-induced HCC rats, the number of atypical cells and inflammatory cells decreased significantly; the serum alpha fetoprotein level and Fisher’s ratio tended to return to normal; the median survival time increased from 36 to 96 days (*p* = 0.08). In addition, serum malondialdehyde, superoxide dismutase, and glutathione activity nearly returned to the level of the healthy control group.

**Conclusion:** The elevation of serum uric acid implies a risk of poor survival in advanced HCC patients and Febuxostat can reduce the generation of reactive oxygen species, thereby playing a role in delaying the progression of liver cancer.

## Introduction

The etiology of hepatocellular carcinoma (HCC) mainly includes viral infections, such as hepatitis B and C, and the ingestion of toxic substances (Singal and El-Serag, 2015; Mak et al., 2018). HCC is a serious threat to human health. According to epidemiological statistics, HCC has the second highest mortality rate among all types of malignant tumors, and the number of patients with HCC is increasing, which has gradually become a major problem in public health (Bray et al., 2018). Despite the continuous improvement of treatment regimens, the long-term prognosis of HCC patients is poor, and the 5-years survival rate of HCC patients in China is only 14.1% (Allemani et al., 2018).

Ten purine metabolites were previously determined in the serum of patients with HCC. We found that the serum levels of xanthine, guanine, hypoxanthine, xanthosine, and guanosine were significantly altered in HCC patients compared with healthy controls. In particular, increased levels of serum uric acid were observed in the HCC group compared with chronic hepatitis B-infected individuals and healthy controls (Zhang et al., 2019). In the human body, uric acid is the end product of the purine metabolism pathway, which is produced by xanthine and hypoxanthine under the action of xanthine oxidoreductase (XOR). XOR is an important rate-limiting enzyme in purine nucleic acid catabolism in cells or *in vivo* (Bulkley, 1993; Xu et al., 2016). It has two different forms, namely, xanthine oxidase (XOD) and xanthine dehydrogenase (XDH) (Grum et al., 1986). Our experiment also showed that the XOD activity of the HCC group was significantly higher than that of the healthy control group (Zhang et al., 2019). Recently, Masamichi Hayashi revealed that high uric acid could be a significant risk factor of activating hepatocarcinogenesis (Hayashi et al., 2020). A retrospective study of patients undergoing surgery for renal cell carcinoma (RCC) revealed that increasing serum uric acid levels are negatively associated with survival in RCC (Yim et al., 2019). However, at present, little is known about the relationship between serum uric acid levels and survival time in HCC patients and whether inhibiting uric acid production can delay the progression of HCC.

Here, the relationship between the survival time of 288 patients with advanced HCC and their serum uric acid levels was analyzed retrospectively. A diethylnitrosamine (DEN)-induced HCC rat model provides an ideal animal model for studying the occurrence and development of liver cancer and drug interventions (Alzahrani et al., 2018; Singh et al., 2018). Therefore, the changes of purine metabolism in HCC rats induced by DEN were verified by an established UPLC-MS/MS method. On this basis, febuxostat, a specific inhibitor of XOD, was used to interfere with the HCC rats to understand its effect on natural survival.

## Materials and Methods

### Reagents and Instruments

The reagents and instruments for measuring purine metabolites were described previously (Zhang et al., 2019). The kit for measuring XOD was purchased from Nanjing Jiancheng Bioengineering Institute (Nanjing, China). TRIzol and RNA reverse transcription kit were purchased from TransGen Biotech Co., Ltd (Beijing, China). RIPA buffer, protease inhibitor, and Tris-Tricine-SDS-PAGE kit were purchased from Beijing Solarbio Science and Technology Co., Ltd. (Beijing, China).

### Retrospective Analysis of the Correlation Between Survival Time and Serum Uric Acid Levels of HCC Patients

A total of 288 HCC patients with TNM stage III/Ⅳ admitted to the Second Affiliated Hospital of Nanchang University and the Affiliated Cancer Hospital of Nanchang University from February 2016 to July 2018 were selected for analysis. The patients were diagnosed and treated in accordance with the Guidelines for the Diagnosis and Treatment of Primary Liver Cancer (2016 edition). The inclusion criteria were the follows: 1) patients were diagnosed with pathologically confirmed HCC in TNM stage III/Ⅳ. 2) Patients have completed medical record of history and tests. 3) The patients were followed up through call visits in 3 years. The correlation between serum uric acid levels before treatment and the survival time was analyzed. The risk factors associated with HCC were analyzed by Cox regression method.

### DEN-Induced HCC Rats

Thirty healthy male SD rats (weighing 180–200 g) were randomly divided into the control group (*n* = 10) and DEN group (*n* = 20). The DEN group was injected intraperitoneally with 60.0 mg/kg of DEN every day for 10 weeks, whereas the control group was injected with the same volume of normal saline. Blood samples were taken after fasting for 12 h, and all rats were killed after 14 weeks. A complete liver was cut and immersed in formalin solution for pathological examination. Part of the liver was cut and stored in RNA hold solution, and the rest of the liver was stored at −80°C.

### Determination of Purine Metabolites in Serum and Liver of Rats

The serum sample preparation for UPLC-MS/MS analysis and the detection conditions were described in a previous literature (Zhang et al., 2019). The liver tissue was thawed at room temperature. About 0.3 g of liver tissue was accurately weighed and then placed in a homogenizer. About 5.0 ml of 80% methanol in three times was added for homogenization. The homogenization liquid was collected and centrifuged at 8,000 rpm for 10 min. The supernatant was prepared in accordance with the serum sample preparation for further analysis.

### Determination of XOD Activity and Gene Expression of XDH mRNA in Liver Tissue

The XOD activity in serum and liver tissue was measured in accordance with the instructions of the detection kit. The extraction and reverse transcription of total RNA from the liver were performed in accordance with the instructions of the related kits. Real-time fluorescent quantitative polymerase chain reaction (PCR) was performed using Takara TB Green™ Premix Ex Taq™, and the TB Green chimeric fluorescence method was used for real-time PCR. GAPDH was selected as the internal reference gene. The primer sequence, designed and synthesized by Shanghai Shenggong Bioengineering Co., Ltd. and Hunan Qinke biological Co., Ltd. is shown in Supplementary Table S1.

### Febuxostat Intervention With DEN-Induced HCC Rats

A total of 65 healthy male SD rats (weighing 180–200 g) were selected and divided into three groups, namely, healthy control group (*n* = 5), DEN group (*n* = 29), and DEN + Febu group (*n* = 29). The control group was given 2.0 ml of 0.9% NaCl-injectable solution and 10% Tween 80 (v/v) orally. The DEN group was intraperitoneally injected with DEN (60.0 mg/kg, dissolved in a 0.9% NaCl-injectable solution) and given 2.0 ml of 0.9% NaCl-injectable solution with 10% Tween 80 (v/v) orally. The DEN + Febu group was intraperitoneally injected with DEN (60.0 mg/kg, dissolved in a 0.9% NaCl-injectable solution) and orally administered with febuxostat (20.0 mg/kg, dissolved in a 0.9% NaCl-injectable solution and 10% Tween 80, v/v). At the 14th week of administration, all rats in the healthy control group were sacrificed, and 14 rats in the DEN and DEN + Febu groups were sacrificed. Blood and liver tissues were taken for analysis. The remaining rats were fed, and the natural survival time of the DEN + Febu (*n* = 15) and DEN groups (*n* = 15) was observed.

### Statistical Analysis

The Kaplan–Meier curves was performed by GraphPad Prism 8.0 (GraphPad Software, San Diego, CA, United States). The Cox regression was analyzed by R software (R x64 4.0.3 version). The quantitative data were analyzed using SPSS 23.0 software (IBM Corp. Armonk, NY, United States) with an independent sample *t*-test.

## Results

### Univariant Analysis of Risk Factors for Survival in Advanced HCC Patients

A retrospective analysis was conducted on 288 HCC patients with TNM stage of III/Ⅳ and complete clinical examination and follow-up survival data. Among them, there were 232 males and 56 females, aged from 11 to 88 years old, with an average age of 57.1 years old and survival time of 205 days. Univariant analysis revealed that serum total bilirubin, direct bilirubin, glutamate transpeptidase, nucleotidase, total bile acid, cholinesterase, fucosidase, cystatin C, creatinine and uric acid were statistically significant risk factors for survival in advanced HCC patients (Supplementary Table S2).

### Negative Correlation Between Serum Uric Acid Levels and Survival in Patients With Advanced HCC

**FIGURE 1:**
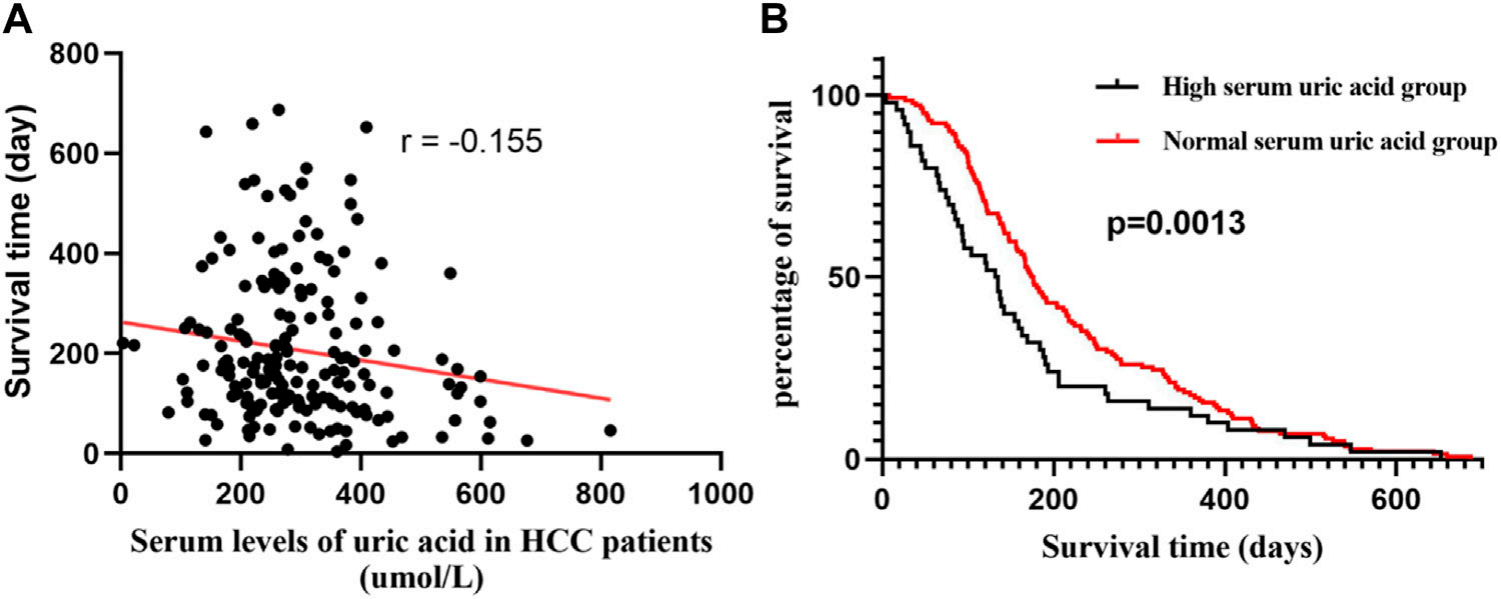
The results of retrospective analysis in advanced HCC patients. **(A)** Correlation of serum uric acid and survival time. **(B)**Kaplan–Meier curves of high and normal serum uric acid (Gehan-Breslow-Wilcoxon test, *p* = 0.0013).

Correlation analysis was conducted between serum uric acid levels at admission and survival days. The results showed that the serum uric acid levels were significantly negatively correlated with survival days (*r* = −0.155, Figure 1). Furthermore, all patients were grouped into high uric acid group (>360 μmol/L, *n* = 80) and normal serum uric acid group (<360 μmol/L, *n* = 208) groups at the pretreatment time point. The survival curve analysis showed that the median survival time of the high uric acid group was 133.5 days, and that of the normal serum uric acid group was 176.0 days. Gehan-Breslow-Wilcoxon test showed significant difference between the two groups (*p* = 0.0013), and the hazard ratio was 1.41 (95% CI of ratio: 0.9923–2.014). These data suggest that lowering the uric acid levels may improve the prognosis and prolong the survival of patients.

### Contents of Purine Metabolites in Serum and Liver Samples of HCC Rats Induced by DEN

The concentrations of purine metabolites in serum and liver were expressed as mean ± S.E.M (Table 1). Compared with the control group (*n* = 10), the DEN group (*n* = 20) showed a significantly downregulated serum nucleic acid xanthine content (-31.17%) and significantly upregulated contents of hypoxanthine (+56.4), guanine (+520%) and uric acid (+20.7%). The liver of HCC rats exhibited significantly upregulated contents of xanthine (+119.33%), hypoxanthine (+41.03%), guanine (+127.11%), guanine nucleoside (+42.62%) and uric acid (+83.7%), and significantly downregulated content of uridine (−29.56%).

**TABLE 1:** Absolute quantitation of purine metabolites in serum and liver of rats (mean ± S.E.M).

Metabolites	Levels in serum (μg/ml)		Levels in liver (mg/g)	
	Control group (*n* = 10)	DEN group (*n* = 20)	Control group (*n* = 10)	DEN group (*n* = 20)
Xanthine	0.045 ± 0.014	0.031 ± 0.003*	41.68 ± 3.92	91.44 ± 4.35**
Adenine	1.41 ± 0.350	1.675 ± 0.232	91.45 ± 5.1	129.59 ± 2.22**
Guanine	0.030 ± 0.002	0.186 ± 0.042**	3.38 ± 0.68	4.36 ± 0.07*
Hypoxanthine	2.921 ± 1.22	4.537 ± 1.035*	3.80 ± 0.85	8.64 ± 1.73**
Xanthosine	0.07 ± 0.0005	0.071 ± 0.0004	29.98 ± 6.16	28.00 ± 3.25
Adenosine	3.812 ± 0.044	4.662 ± 0.158**	0.065 ± 0.03	0.010 ± 0.01
Guanosine	0.015 ± 0.001	0.016 ± 0.0009	0.100 ± 0.03	0.028 ± 0.002
Inosine	4.320 ± 0.165	4.62 ± 0.158	16.28 ± 3.57	23.22 ± 0.88*
Uridine	1.286 ± 0.080	1.369 ± 0.088	17.69 ± 3.50	12.46 ± 2.2*
Uric acid	30.03 ± 0.41	36.25 ± 0.735*	56.52 ± 13.73	103.8 ± 14.92*

Note: * represent significant differences DEN group from the Control group *p < 0.05, **p < 0.01.

### Alterations of XOD Activity and Uric Acid Synthesis Gene in HCC Rats

**FIGURE 2:**
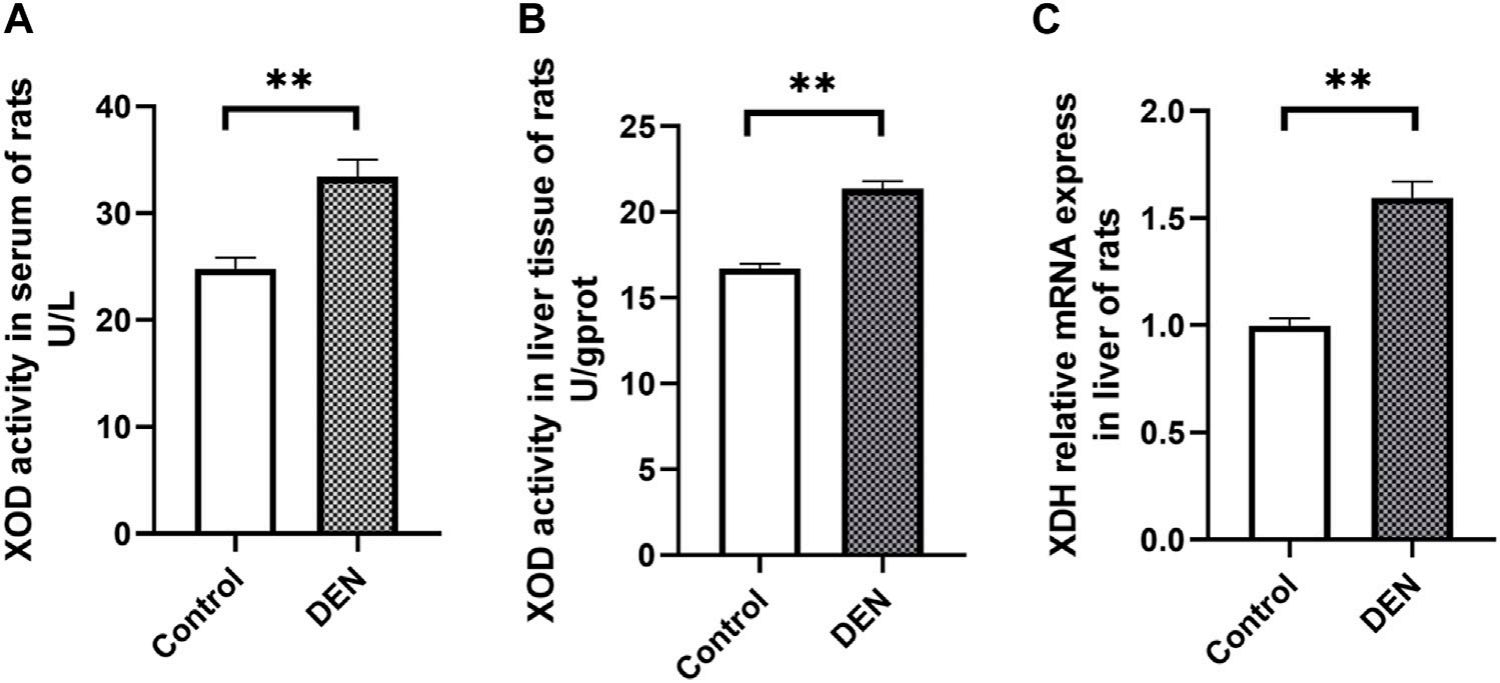
The XOD activity in serum **(A)**, liver tissues **(B)** and the XDH mRNA level **(C)** in liver of healthy (control) and HCC (DEN) rats. **represent significant differences of DEN group (*n* = 20, mean ± S.E.M) from the control group (*n* = 10, mean ± S.E.M, **p < 0.01).

The XOD activity in serum and liver samples of rats was significantly increased in the DEN group compared with the control group (Figures 2A,B). As shown in Figure 2C, the XDH mRNA expression in the liver of rats was significantly upregulated in the DEN group compared with the control group.

### Effect of Febuxostat on Pathological and Serological Indicators of HCC Rats Induced by DEN

**FIGURE 3:**
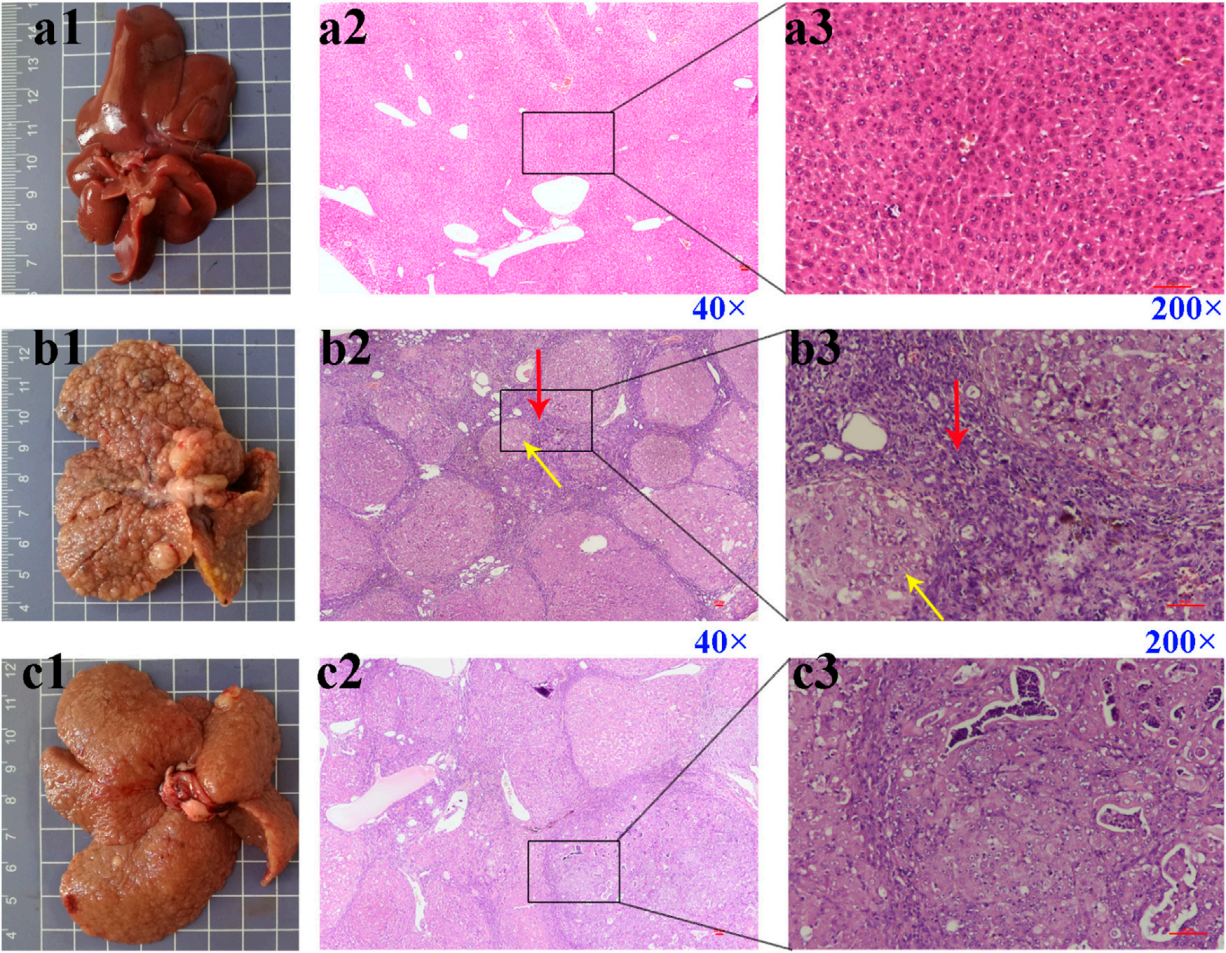
Pictures (scale bar, cm) and H&E staining images of rat liver tissue. **(a1–a3)** are the control group; **(b1–b3)** are the DEN group; **(c1–c3)** are the DEN + Febu group. Red arrow: proliferative fibrous connective tissue; Yellow arrow: pseudolobuli formation.

The liver of rats in the control group was smooth and soft (Figure 3a1). The liver surface of rats in the DEN (Figure 3b1) and DEN + Febu (Figure 3c1) groups showed a large number of white cancer nodules, and the liver was enlarged, with rough surface and slightly hard texture. The H&E staining of the rat liver in the DEN group showed marked fibrous septum and pseudolobule formation (Figures 3b2,b3). Whereas, The H&E image of DEN + Febu group showed a decrease in the number of pseudolobule, proliferative fibrous connective tissue, and choronic inflammation cell infiltration (Figures 3c2,c3).

**FIGURE 4:**
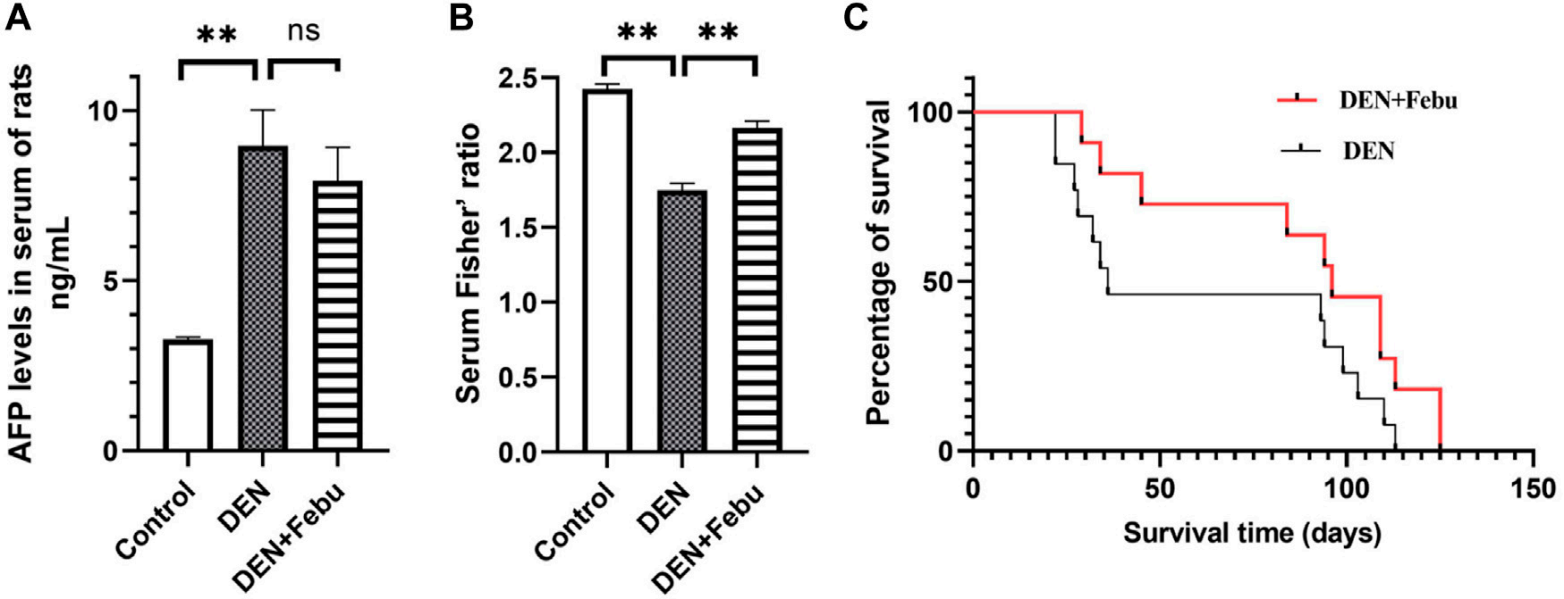
Serum AFP level **(A)**, Serum Fisher’ ratio in rats of control (n = 5), DEN (n = 14) and DEN + Feu (*n* = 14) group **(B)** and the survival curve (**C**, log-rank Mantel–Cox test, *p* = 0.0802) of HCC rats induced by DEN (*n* = 15) and those treated by Febuxostat (*n* = 15). **represent significant differences between groups (mean ± S.E.M, **p < 0.01).

Figure 4A shows that the serum alpha fetoprotein (AFP) of rats in the DEN group was significantly higher than that in the healthy control group (3.277 ± 0.068 vs. 8.962 ± 1.051 ng/ml). After febuxostat intervention, the serum AFP level showed a decreasing trend (7.937 ± 0.973). Furthermore, the serum levels of leucine, isoleucine, valine, phenylalanine, and tyrosine were determined by an established HPLC-UV method, and Fisher’s ratio was calculated (Yang et al., 2018). Our results showed that Fisher’s ratio was significantly decreased in the DEN group compared with the healthy control group and significantly increased in the DEN + Febu group compared with the DEN group (Figure 4B).

### Febuxostat Prolonged the Survival of HCC Rats

The median survival days of DEN + Febu rats increased from 36 to 96 days (*n* = 15, log-rank Mantel–Cox test) with a hazard ratio of 1.93 (95% confidence interval: 0.845–4.393, *p* = 0.08) (Figure 4C).

### Effect of Febuxostat on the Antioxidant Capacity of HCC Rats Induced by DEN

Compared with the control group, the malondialdehyde (MDA) content in serum and liver tissues of rats in the DEN group increased significantly. After febuxostat intervention, the MDA content returned to the level of the healthy control group. Meanwhile, the activities of superoxide dismutase (SOD) and glutathione (GSH) in serum and liver tissues of rats in the DEN group decreased significantly. After febuxostat intervention, the activities of SOD and GSH in serum returned to the level of the healthy control group (Table 2).

**TABLE 2:** MDA, SOD, and GSH activity in serum and liver of rats (mean ± S.E.M).

		Control (*n* = 5)	DEN (*n* = 14)	DEN + Febu (*n* = 14)
Serum	MDA (nmol/ml)	15.37 ± 1.31	25.81 ± 4.13^a^	13.39 ± 1.30^b^
	SOD (U/ml)	591.07 ± 16.78	562.03 ± 14.34^a^	615.97 ± 13.53^b^
	GSH (μmol/ml)	22.3 ± 1.13	13.4 ± 1.23^a^	18.2 ± 2.13^b^
Liver tissue	MDA (nmol/mgprot)	2.64 ± 0.30	7.12 ± 0.57^a^	4.34 ± 0.59^b^
	SOD (U/mgprot)	846.47 ± 26.06	724.20 ± 10.75^a^	704.53 ± 18.28
	GSH (μmol/gprot)	7.81 ± 0.45	4.45 ± 0.23^a^	6.74 ± 0.46^b^

MDA, malondialdehyde; SOD, superoxide dismutase; GSH, glutathione.

a Note: represent significant differences between the DEN group and the Control group (*p < 0.05).

b represent significant differences between the DEN + Febu group and the DEN group (#*p* < 0.05).

## Discussion

Researchers found that serum uric acid is significantly increased in patients with tumors and showed a significant upward trend with tumor progression; thus, it can be used as a biomarker for the diagnosis and prognosis of head and neck squamous cell carcinoma (Dhankhar et al., 2011). Uric acid can be used as a biomarker for the diagnosis and prognosis of pancreatic cancer, and increased uric acid levels are closely related to increased tumor mortality (Stotz et al., 2014). High serum uric acid is also a significant risk factor of activating hepatocarcinogenesis (Hayashi et al., 2020). Our previous study showed that the serum levels of inosine, xanthine, and guanosine in HCC patients were significantly decreased, and those of hypoxanthine, xanthine, guanine, uric acid and XOD activity were significantly increased compared with those in healthy people (Zhang et al., 2019). To date, little is known about the relationship between serum uric acid levels and survival time in HCC patients. Here, the retrospective analysis of 288 advanced HCC patients showed that the serum uric acid levels were statistically significant risk factors for survival and negatively correlated with the survival time (*r* = −0.155), as well as high uric acid levels corresponded to shorter survival time, suggesting that the accumulation of uric acid in the body may lead to liver cancer progression and shorten the survival time of patients.

The establishment of a liver cancer animal model is a prerequisite for the study of liver cancer pathogenesis, pharmacodynamic evaluation, and mechanism of action. The chemical induction method is widely used due to its convenience, low price and the occurrence of liver cancer is similar to that of human beings, with DEN exhibiting the good induction effect in rats (Kumar and Vijayalakshmi, 2015) and mice (Sharma et al., 2012). Here, our results showed that compared with the control group, the serum contents of hypoxanthine, guanine, and uric acid and the XOD activity were significantly upregulated, which were consistent with the changes in HCC patients, indicating that DEN-induced HCC rats might simulate the changes of purine metabolism in HCC patients.

XOD and XDH are transcriptional products of the same gene at p22 site of human chromosome 2, in which XDH is the transcriptional product and XOD is transformed from XDH (Minoshima et al., 1995). Thus, we further determined the expression of XDH mRNA in rat liver. The results of Q-PCR showed that the expression of XDH mRNA was significantly upregulated, with increased XOD activity in DEN-induced HCC rats, suggesting that the HCC could lead to increased uric acid production.

Febuxostat is a specific inhibitor of XOD, which can inhibit the production of uric acid *in vivo* by inhibiting the activity of this enzyme. The potency (EC50) of it was observed to be 128 ng/ml (Leander et al., 2021). The blood concentration of febuxostat was estimated to be approximately 125 ng/ml at 19 h after oral administration by the pharmacokinetic investigation in rats (20 mg/kg) (Sahu et al., 2014). Thus, the steady-state blood concentration of febuxostat would be further higher than EC50 after multiple-dosage regimen to achieve the desired pharmacological effects. Our results showed that after febuxostat intervention, the liver pathological sections of rats (DEN + Febu group) showed a lower degree of liver lesions than those of the DEN group, and the serum AFP content decreased significantly and returned to normal levels. In addition, when liver diseases occur, the metabolism of aromatic amino acids is blocked, leading to the decrease of Fisher’s ratio of amino acids in serum and liver (Ishikawa, 2012; Tada et al., 2015; Kinny-Köster et al., 2016). Our results also showed that Fisher’s ratio in the DEN group was significantly decreased, whereas that in the DEN + Febu group was significantly increased (close to the level of the healthy control group). These results suggested that inhibiting uric acid production could significantly delay the progression of liver cancer.

Importantly, we found that the median survival days increased from 36 days of DEN group to 96 days of DEN + Febu group. Similar to our experimental results, Konishi et al. (Konishi et al., 2015) established a rat tumor cachexia model by using ascitic liver cancer cells, a common cancer complication caused by cancer and other serious chronic consumptive diseases. After daily intragastric administration of febuxostat (5 mg/kg/day), the 17-days survival rate of rats in the febuxostat-group increased from 20% in the placebo group to 59%, and the median survival days increased from 14 to 17 days. Furthermore, Springer et al. previously confirmed that allopurinol could significantly improve the survival time of tumor cachexia rats, and the mechanism was involved in reactive oxygen species (ROS)-related anti-catabolism (Springer et al., 2012). Due to the increase of XOR activity under tumor conditions, a large amount of ROS such as hydrogen peroxide (H_2_O_2_) and superoxide (O_2_^−^) are generated while uric acid is produced. Therefore, we speculated that febuxostat could delay the progression of liver cancer and prolong the survival period, which might be related to the decreased ROS levels *in vivo*.

By balancing the production and removal of ROS, normal cells stabilize the content of ROS within a normal range. SOD is a kind of metalloproteinase that can maintain the balance between the production and scavenging of oxygen free radicals in the body (Czeczot et al., 2006; Cerdá-Bernad et al., 2020). Our results showed that the activities of SOD and GSH in the DEN group were significantly downregulated, and febuxostat intervention could reverse this trend. In addition, MDA is one of the end products of lipid peroxidation in the body, which can promote the occurrence and development of tumors. In tumor and vascular diseases, the MDA levels are significantly increased (Zińczuk et al., 2019; Cerdá-Bernad et al., 2020; Zhevak et al., 2020). Our results also showed that the MDA levels in serum and liver tissue of the DEN group were significantly upregulated, and febuxostat reversed the upregulation of MDA to the level of the healthy control group. Therefore, inhibiting XOD to reduce the production of ROS could delay the progression of HCC.

## Conclusion

The results of this study show that the elevation of serum uric acid implies a risk of poor survival in advanced HCC patients, and purine metabolism were significantly altered in DEN-induced HCC rats. Our findings suggest that inhibition of XOD could significantly delay the progression of liver cancer and result in prolonged survival time through reducing ROS production, which has high clinical translational value.

## Data Availability

The raw data supporting the conclusions of this article will be made available by the authors, without undue reservation.

## Abbreviations

AFP, Alpha-Fetoprotein; AST, aspartate transaminase; DEN, diethylnitrosamine; HCC, hepatocellular carcinoma; H2O2, hydrogen peroxide; GSH, glutathione; MDA, malondialdehyde; OS, overall survival; PCR, polymerase chain reaction; RCC, renal cell carcinoma; SOD, superoxide dismutase; UPLC-MS/MS, ultra performance liquid chromatography/tandem mass spectrometry; XDH, xanthine dehydrogenase; XOD, xanthine oxidase; XOR, xanthine oxidoreductase.
