# Supplementary material for: Elevated Serum Uric Acid is Associated With Poor Survival in Advanced HCC Patients and Febuxostat Improves Prognosis in HCC Rats
Source: Front Pharmacol. 2021 Nov 11;12:778890. doi: 10.3389/fphar.2021.778890 (PMC8632057; doi:10.3389/fphar.2021.778890)
Supplement: Supplementary file 1 [file DataSheet1.docx]

**sTable 1** The information of gene transcription analyses and Ct values (mean±S.E.M) for Q-PCR.

| Gene | Primer Sequences | Product size | Ct value  Control group | Ct value  DEN group |
| --- | --- | --- | --- | --- |
| GAPDH | Forward: ACAGCAACAGGGTGGTGGAC | -- | 16.0±0.39 | 16.2±0.25 |
|  | Reverse: TTTGAGGGTGCAGCGAACTT |  |  |  |
| XDH | Forward: CTGTGGAGAAGGTGGCTGTG | 124 | 27.2±0.04 | 26.6±0.10 |
|  | Reverse: AACATGGTGCAAGGAGCAGA |  |  |  |

**sTable 2.** Univariant analysis of risk factors for survival in advanced HCC patients.

|  | HR | C.l.( 95%) | p value |
| --- | --- | --- | --- |
| Gender | 1.17 | 0.88-1.56 | 0.27 |
| Age | 0.79 | 0.62-1.01 | 0.056 |
| Total bilirubin | 1.35 | 1.05-1.73 | 0.019 |
| Direct bilirubin | 1.31 | 1.02-1.69 | 0.038 |
| Glutamate transpeptidase | 1.49 | 1.16-1.92 | 0.002 |
| Nucleotidase | 1.44 | 1.13-1.84 | 0.004 |
| Total bile acid | 1.35 | 1.05-1.72 | 0.018 |
| Cholinesterase | 0.64 | 0.51-0.81 | 0.0 |
| Fucosidase | 1.19 | 0.92-1.53 | 0.188 |
| Cystatin C | 1.34 | 1.03-1.73 | 0.026 |
| Urea nitrogen | 0.93 | 0.65-1.34 | 0.704 |
| Creatinine | 1.66 | 1.15-2.39 | 0.006 |
| Uric acid | 1.46 | 1.04-2.05 | 0.028 |
| AFP | 1.11 | 0.87-1.41 | 0.398 |

HR, hazard ratio; C.I., confidence interval.
